# Global and regional estimates of vaccine-associated herpes zoster and their related vaccines from 1969 to 2023

**DOI:** 10.1038/s41598-025-98106-9

**Published:** 2025-04-17

**Authors:** Jinyoung Jeong, Hyesu Jo, Yejun Son, Jaeyu Park, Jiyeon Oh, Sooji Lee, Yi Deun Jeong, Kyeongmin Lee, Hyeon Jin Kim, Hayeon Lee, Soeun Kim, Yesol Yim, Masoud Rahmati, Jiseung Kang, Raphael Udeh, Damiano Pizzol, Lee Smith, Jiyoung Hwang, Dong Keon Yon

**Affiliations:** 1 Department of Medicine, Kyung Hee University College of Medicine, Seoul, South Korea; 2 Center for Digital Health, Medical Science Research Institute, Kyung Hee University Medical Center, Kyung Hee University College of Medicine, 23 Kyungheedae-ro, Dongdaemun-gu, Seoul, 02447 South Korea; 3 Department of Regulatory Science, Kyung Hee University, Seoul, South Korea; 4 Department of Precision Medicine, Kyung Hee University College of Medicine, Seoul, South Korea; 5 Department of Electronics and Information Convergence Engineering, Kyung Hee University, Yongin, South Korea; 6 Health Service Research and Quality of Life Center (CEReSS), Assistance Publique-Hôpitaux de Marseille, Aix-Marseille Université, Marseille, France; 7 Department of Physical Education and Sport Sciences, Faculty of Literature and Human Sciences, Lorestan University, Khoramabad, Iran; 8 Department of Physical Education and Sport Sciences, Faculty of Literature and Humanities, Vali-E-Asr University of Rafsanjan, Rafsanjan, Iran; 9 School of Health and Environmental Science, College of Health Science, Korea University, Seoul, South Korea; 10 Department of Health and Safety Convergence Science, Korea University Graduate School, Seoul, South Korea; 11 School of Life Sciences, Faculty of Science, University of Technology Sydney, Ultimo, Australia; 12 Health Unit, Eni, San Donato Milanese, Italy; 13 Health Unit, Eni, Maputo, Mozambique; 14 Centre for Health, Performance and Wellbeing, Anglia Ruskin University, Cambridge, CB1 1PT UK; 15 Department of Pediatrics, Kyung Hee University College of Medicine, 23 Kyungheedae-ro, Dongdaemun-gu, Seoul, 02447 South Korea

**Keywords:** Global, Herpes zoster, Pharmacovigilance, Vaccines, Health care, Medical research

## Abstract

Vaccine-induced immunosuppression can reactivate the varicella-zoster virus, potentially leading to the development of herpes zoster. However, the literature on this topic is inconsistent, resulting in limited clarity. Therefore, we aimed to enhance our understanding of vaccine-associated herpes zoster and establish guidelines for future research, utilizing a global database to improve global public health. We investigated vaccine-associated adverse events in herpes zoster using reports (~ 13 million reports) from the WHO international pharmacovigilance database. Data were analyzed for the global number of reports, reported odds ratios (ROR), and information components (IC) to determine the potential association between 18 vaccines and vaccine-associated herpes zoster reports in nearly 170 countries and territories from 1969 to 2023. Of 7,805,380 vaccine-associated adverse events, there were 51,985 herpes zoster reports. Vaccine-associated herpes zoster showed the highest strength of association with COVID-19 mRNA vaccines (ROR, 11.85 [95% CI, 11.70-12.01]; IC, 2.74 [IC_0.25_, 2.72]), followed by encephalitis (ROR, 4.07 [95% CI, 3.37–4.92]; IC, 2.00 [IC_0.25_, 1.68]), influenza (ROR, 3.44 [95% CI, 3.28–3.62]; IC, 1.77 [IC_0.25_, 1.69]), and ad5-vectored COVID-19 vaccines (ROR, 3.05 [95% CI, 2.97–3.14]; IC, 1.54 [IC_0.25_, 1.50]). The ROR and IC of vaccine-associated herpes zoster in males (ROR, 7.94 [95% CI, 7.80–8.08]; IC, 2.47 [IC_0.25_, 2.45]) and females (ROR, 6.71 [95% CI, 6.62–6.80]; IC, 2.30 [IC_0.25_, 2.28]). The ROR and IC increased with advancing age. Our findings emphasize the need to consider the immune status of vaccine recipients and to implement appropriate compensation and management manuals for vaccine-associated herpes zoster.

**Supplementary Information:**

The online version contains supplementary material available at 10.1038/s41598-025-98106-9.

## Introduction

The global health environment has seen notable advancements in vaccine development, especially in response to the COVID-19 pandemic^1,2^. These vaccines have been pivotal in global public health strategies, effectively reducing disease burden and mortality rates^3^. Nevertheless, similar to several medical interventions, vaccines may evoke adverse events^4^. One of the side effects following vaccination is a temporary decrease in immune response due to the immune system’s reaction to the vaccine^5^. This immunosuppression can also lead to the reactivation of the varicella zoster virus (VZV), potentially resulting in the onset of the zoster^6^.

While these vaccine-associated herpes zoster does not have a high mortality^7^, it can pose a serious threat to the quality of life with painful rashes and nerve pain, as well as serious complications such as encephalitis^8^. Accordingly, many previous studies suggested that various vaccines can cause the onset of zoster, with numerous studies presenting that COVID-19 vaccines may trigger zoster^9^. However, there has been a lack of long-term research on the effects of vaccines on zoster, and studies exploring how vaccine side effects vary by sex and age have also not been conducted.

Therefore, we aimed to investigate the potential association of various vaccines with herpes zosters using the global pharmacovigilance database. Based on the findings of this study, we tried to enhance understanding of vaccine-associated herpes zoster, provide guidelines for future research, and propose appropriate policies to address vaccine-associated herpes zoster.

## Methods

### Data sources

Our study aimed to investigate the potential adverse effects associated with vaccines, focusing specifically on herpes zoster, by utilizing the World Health Organization (WHO) international pharmacovigilance database. WHO international pharmacovigilance database is the comprehensive global repository that aggregates reports of potential adverse drug reactions^10^. Since its inception in 1969, it has participated in the WHO International Drug Monitoring Program, containing over ~ 13 million reports associated with suspected adverse drug reactions reported by countries actively participating in the program^4,10,11^.

Systematic data collection is ongoing, and our dataset encompasses data collected until June 2023^11,12^. This extensive database comprises information on over 25,000 different pharmaceuticals, providing a comprehensive foundation for the safety evaluation of medications and vaccines^13^. The confidential use of this data was authorized by the Institutional Review Board of Kyung Hee University. Ethical approval and consent were not required as this study was based on publicly available data.

### Selection of cases

Data associated with vaccines were collected between 1969 and June 2023. The vaccines were categorized into eighteen different groups: (1) anthrax, (2) cholera, (3) diphtheria, tetanus toxoids, pertussis, polio, and *Hemophilus influenza*type b [DTaP-IPV-Hib], (4) meningococcal, (5) pneumococcal, (6) tuberculosis, (7) typhoid, (8) encephalitis (excluding live attenuated vaccines), (9) influenza, (10) hepatitis A, (11) hepatitis B, (12) measles, mumps, and rubella (MMR), (13) rotavirus diarrhea, (14) papillomavirus, (15) COVID-19 mRNA, (16) Ad5-vectored COVID-19, (17) inactivated whole-virus COVID-19, and (18) others (brucellosis, dengue vaccines, Ebola, leptospirosis, plague, rabies, smallpox, tuberculosis, typhus, and yellow fever vaccines)^4^. In this study, we systematically collected and analyzed adverse event reports from non-duplicated sources worldwide, utilizing the Medical Dictionary for Regulatory Activities version 26.0^11,14^. Our focus was primarily on the terms listed in **Table ****S1**, with a particular emphasis on herpes zoster as reported adverse reactions from global vaccination events. In compliance with the WHO guidelines for causality assessment, our analysis included only those vaccines identified as ‘suspected’ of having a disproportionate association with herpes zoster.

### Data collection

To meticulously investigate cases of suspected zoster linked to vaccination, our study thoroughly compiled individual case safety reports acquired from various sources in the post-marketing setting, including patients, healthcare professionals, and pharmaceutical companies, which served as the principal data source for our analysis. The dataset included the following: patient demographics (i.e., age [0 to 11, 12 to 17, 18 to 44, 45 to 64, ≥ 65 years, and unknown] and sex), administrative information (i.e., reporting regions, reporting years, and reporter qualifications [health and non-health professionals, and unknown]). Information on vaccinations (i.e., type and single suspected vaccine), adverse effects, fatal outcomes (recovered/recovering, fatal, and unknown), and time to onset were also included^15^. Every voluntarily submitted report mentioned at least one vaccine suspected of being linked to herpes zoster adverse event.

### Statistical analysis

An analysis of disproportionality was carried out for each vaccine listed in the WHO international database to investigate any possible associations with zoster incident reports^4^. The analysis employed two well-established pharmacovigilance metrics: the information component (IC) and the reporting odds ratio (ROR)^4^. The IC is a Bayesian measure employed by the WHO for disproportionality analyses in pharmacovigilance. It is designed to detect potential safety signals by comparing the observed number of adverse drug reaction (ADR) reports with the number expected, based on overall reporting rates in the database. The Bayesian model incorporates prior information and accounts for uncertainty, enhancing the robustness of signal detection^16^. In this study, the IC was calculated by comparing the rate of vaccine associated herpes zoster to the cumulative rate of this event for all other medications reported in our database. The method incorporates the expected number of reports and the observed instances for each vaccine-adverse event pair. IC_0.25_ represents the lower boundary of the 95% confidence interval, and IC_0.25_> 0.00 is generally considered statistically significant signals^17^.

The ROR is a frequentist-based measure commonly employed in pharmacovigilance for disproportionality analyses. Similar to the IC, it aims to identify potential safety signals by comparing the observed frequency of a specific ADR with the frequency expected based on overall reports. In this method, the odds of reporting a particular ADR for a given drug are compared to the odds of reporting the same ADR for all other drugs. We calculated the ROR by examining the rate of vaccine associated herpes zoster relative to all other reported events for that vaccine and then comparing these data with comparable data for other medications in our database. ROR values greater than 1.00—along with 95% confidence intervals that exclude 1.00—are generally interpreted as potential signals warranting further investigation^18^. The advantage of ROR is its straightforward calculation and interpretation, which facilitates large-scale comparative analyses of drug-event associations^19^. Statistical significance was taken into consideration when the two-sided p-value was less than 0.05^20^. All analyses were conducted using SAS software (version 9.4; SAS Institute, Cary, NC, USA).

## Results

### Overall analysis

**Fig. 1:**
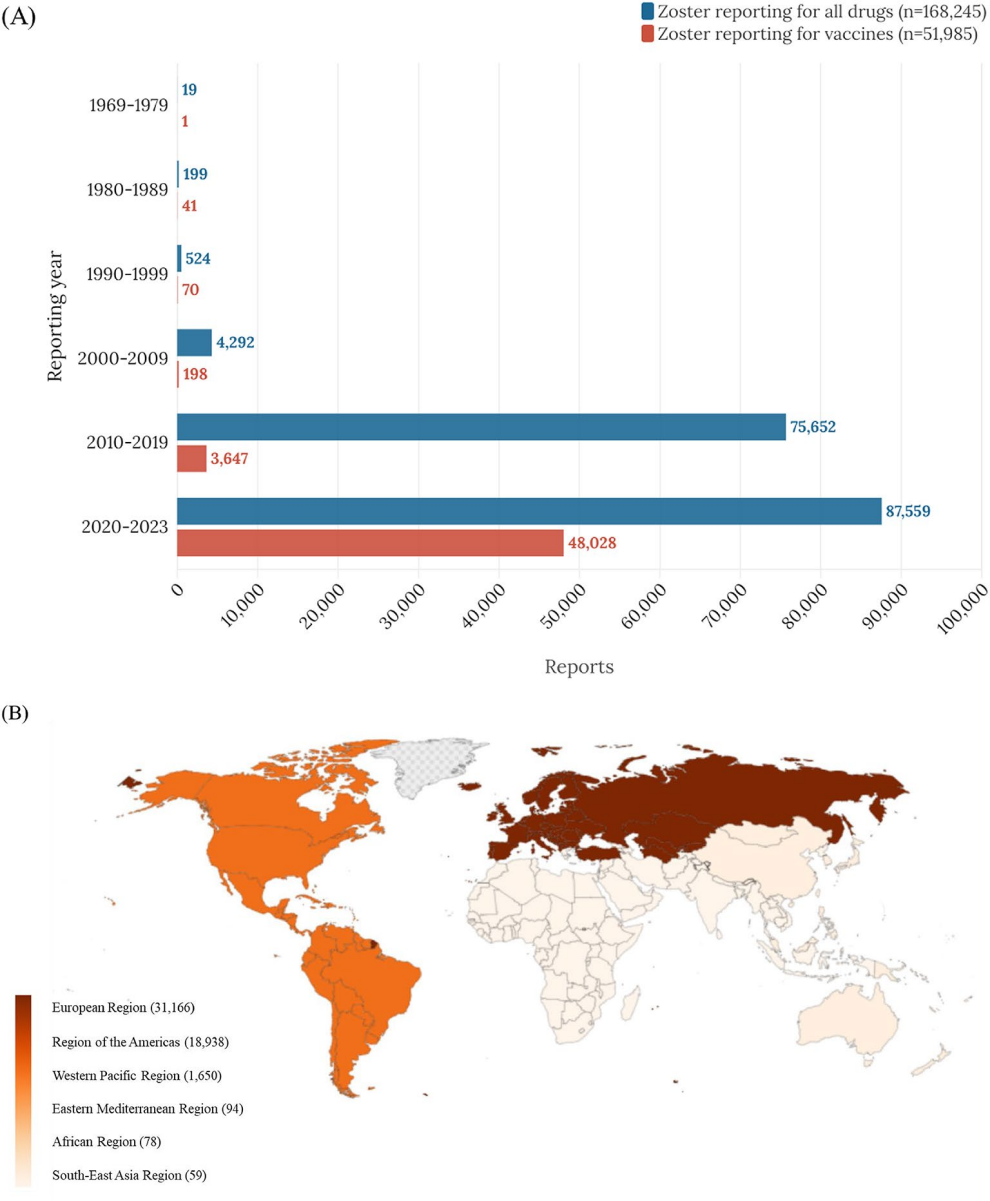
Temporal trends (**A**) and global distribution (**B**) of vaccine-associated herpes zoster adverse events by continent.

Based on the WHO international pharmacovigilance database, a study from 1969 to 2023 included 7,805,380 total vaccine-associated adverse events, containing 51,985 vaccine-associated herpes zoster reports. The reports of vaccine-associated herpes zoster has been steadily increasing since 1969, experienced a sharp rise after 2011, and exploded in number after 2020 (Table 1). The world can be divided into six geographical regions (Fig. 1), with the European region accounting for 59.95% and the region of the Americas for 36.43% of all cases of vaccine-associated herpes zoster, followed by the Western Pacific region (3.17%), African region (0.15%), Eastern Mediterranean region (0.18%), and Southeast Asia region (0.11%). The COVID-19 mRNA vaccines exhibited the most significant association with vaccine-associated herpes zoster (79.37%), followed by ad5-vectored COVID-19 vaccines (10.93%) and influenza vaccines (3.1%). The prognosis for vaccine-associated herpes zoster disease was good for almost all individuals, with only 0.06% fatalities (Table 1).

**Table 1:** Baseline characteristics of reports on vaccine-associated herpes zoster adverse events. (*n* = 51,985).

Variables		Number (%)
Region reporting	African region	78 (0.15)
	Region of the Americas	18,938 (36.43)
	Southeast Asia region	59 (0.11)
	European region	31,166 (59.95)
	Eastern Mediterranean region	94 (0.18)
	Western Pacific region	1,650 (3.17)
Reporting year	1969–1979	1 (0.00)
	1980–1989	41 (0.08)
	1990–1999	70 (0.13)
	2000–2009	198 (0.38)
	2010–2019	3,647 (7.02)
	2020–2023	48,028 (92.39)
Reporter qualification	Health professional	9,944 (19.13)
	Non-health professional	22,989 (44.22)
	Unknown	19,052 (36.65)
Sex	Male	18,275 (35.15)
	Female	33,142 (63.75)
	Unknown	568 (1.09)
Age, years	0 to 11	1,777 (3.42)
	12 to 17	779 (1.50)
	18 to 44	11,021 (21.20)
	45 to 64	16,912 (32.53)
	≥ 65	14,640 (28.16)
	Unknown	6,856 (13.19)
TTO, days	Median days (IQR)	1 (1–1)
Drug class	Anthrax vaccines	33 (0.06)
	Cholera vaccines	1 (0.00)
	DTaP-IPV-Hib vaccines	820 (1.58)
	Meningococcal vaccines	118 (0.23)
	Pneumococcal vaccines	467 (0.90)
	Tuberculosis vaccines	5 (0.01)
	Typhoid vaccines	34 (0.07)
	Encephalitis vaccines	108 (0.21)
	Influenza vaccines	1,611 (3.1)
	Hepatitis A vaccines	224 (0.43)
	Hepatitis B vaccines	289 (0.56)
	MMR vaccines	829 (1.59)
	Rotavirus diarrhea vaccines	1 (0.00)
	Papillomavirus vaccines	288 (0.55)
	COVID-19 mRNA vaccines	41,260 (79.37)
	Ad5-vectored COVID-19 vaccines	5,681 (10.93)
	Inactivated whole-virus COVID-19 vaccines	93 (0.18)
	Others^*^	123 (0.24)
Fatal outcomes	Recovered/recovering	33,766 (64.95)
	Fatal	33 (0.06)
	Unknown	18,186 (34.98)
Single drug suspected		51,985 (100.00)

DTaP-IPV-Hib, diphtheria, tetanus toxoids, pertussis, polio, and *Hemophilus influenza* type b; IQR, interquartile range; MMR, measles, mumps, and rubella; TTO, time to onset; WHO, World Health Organization.

^*^Others: brucellosis, dengue vaccines, Ebola, leptospirosis, plague, rabies, smallpox, tuberculosis, typhus, and yellow fever vaccines.

### Analysis of the disproportional vaccine-associated herpes zoster

In terms of vaccines showing an association with vaccine-associated herpes zoster, COVID-19 mRNA vaccines displayed the most highest value of ROR and IC (ROR: 11.85 [95% CI, 11.70–12.01]; IC: 2.74 [IC_0.25_, 2.72]), followed by encephalitis vaccines with a considerable association as well (ROR: 4.07 [95% CI, 3.37–4.92]; IC: 2.00 [IC_0.25_, 1.68]), influenza vaccines (ROR: 3.44 [95% CI, 3.28–3.62]; IC: 1.77 [IC_0.25_, 1.69]), and ad5-vectored COVID-19 vaccines (ROR: 3.05 [95% CI, 2.97–3.14]; IC: 1.54 [IC_0.25_, 1.50]) also impacted.

The ROR and IC for vaccine-associated herpes zoster were 7.94 (95% CI, 7.80–8.08) and 2.47 (IC₀.₂₅, 2.45) in males, and 6.71 (95% CI, 6.62–6.80) and 2.30 (IC₀.₂₅, 2.28) in females. The rate of ROR and IC increased with advancing age, with individuals aged over 65 exhibiting the highest ROR and IC values (Table 2).

**Table 2:** Analysis of subgroups in vaccine-associated herpes zoster adverse events disproportionality.

	Total	Vaccine-associated herpes zoster			IC (IC_0.25_) based on age, years				
		Observed	ROR (95% CI)	IC (IC_0.25_)	0–11	12–17	18–44	45–64	≥ 65
**Total**	7,805,380	51,985	**7.11 (7.03–7.18)**	**2.36 (2.34)**	**0.86 (0.78)**	**1.75 (1.63)**	**2.27 (2.24)**	**2.73 (2.71)**	**3.13 (3.10)**
Sex difference									
Male	2,875,119	18,275	**7.94 (7.80–8.08)**	**2.47 (2.45)**	**0.96 (0.85)**	**1.99 (1.82)**	**2.37 (2.31)**	**2.97 (2.92)**	**3.29 (3.25)**
Female	4,844,872	33,142	**6.71 (6.62–6.80)**	**2.30 (2.28)**	**0.77 (0.65)**	**1.58 (1.42)**	**2.21 (2.17)**	**2.61 (2.58)**	**3.00 (2.97)**
Vaccine types									
Anthrax vaccines	10,007	33	**2.58 (1.83–3.63)**	**1.33 (0.75)**	N/A	N/A	1.83 (1.20)	1.13 (−0.64)	1.52 (−2.26)
Cholera vaccines	2,464	1	0.32 (0.04–2.25)	−1.29 (−5.07)	N/A	N/A	0.01 (−3.77)	N/A	N/A
DTaP-IPV-Hib vaccines	811,899	820	0.79 (0.73–0.84)	−0.34 (−0.46)	**0.17 (0.03)**	**0.63 (0.05)**	**0.62 (0.22)**	**1.20 (0.87)**	**1.39 (0.91)**
Meningococcal vaccines	150,692	118	0.61 (0.51–0.73)	−0.71 (−1.01)	−0.88 (−1.45)	0.38 (−0.20)	**0.91 (0.23)**	1.19 (−0.02)	**2.10 (0.69)**
Pneumococcal vaccines	274,107	467	**1.33 (1.22–1.46)**	**0.41 (0.26)**	**0.51 (0.25)**	0.92 (−1.15)	−0.44 (−1.65)	**1.09 (0.72)**	**0.85 (0.60)**
Tuberculosis vaccines	34,426	5	0.11 (0.05–0.27)	−3.02 (−4.58)	−2.92 (−5.51)	−0.58 (−4.36)	0.91 (−1.68)	N/A	N/A
Typhoid vaccines	17,009	34	**1.56 (1.11–2.19)**	**0.63 (0.06)**	0.07 (−3.71)	N/A	0.72 (−0.22)	**1.32 (0.29)**	**2.02 (0.60)**
Encephalitis vaccines	20,797	108	**4.07 (3.37–4.92)**	**2.00 (1.68)**	0.64 (−0.78)	**2.23 (0.81)**	**2.52 (1.94)**	**2.51 (1.91)**	**2.52 (1.68)**
Influenza vaccines	368,728	1,611	**3.44 (3.28–3.62)**	**1.77 (1.69)**	**0.78 (0.41)**	0.76 (−0.01)	**1.38 (1.15)**	**2.00 (1.85)**	**2.24 (2.11)**
Hepatitis A vaccines	63,160	224	**2.78 (2.43–3.16)**	**1.46 (1.24)**	**2.67 (2.37)**	**1.51 (0.64)**	**1.12 (0.51)**	**1.68 (1.09)**	**2.35 (1.48)**
Hepatitis B vaccines	110,354	289	**2.05 (1.82–2.30)**	**1.03 (0.83)**	**0.72 (0.23)**	**1.49 (0.76)**	**1.67 (1.33)**	**2.13 (1.75)**	**2.76 (1.97)**
MMR vaccines	226,811	829	**2.87 (2.68–3.07)**	**1.51 (1.40)**	**2.49 (2.36)**	**1.52 (0.79)**	**0.75 (0.15)**	**1.70 (0.97)**	**2.79 (1.48)**
Rotavirus diarrhea vaccines	82,458	1	0.01 (0.00–0.07)	−6.15 (−9.93)	−5.03 (−8.82)	N/A	N/A	N/A	N/A
Papillomavirus vaccines	134,319	288	**1.68 (1.49–1.88)**	**0.74 (0.55)**	**1.02 (0.32)**	**1.58 (1.31)**	**1.64 (1.25)**	**3.18 (2.28)**	1.22 (−2.57)
COVID-19 mRNA vaccines	4,009,129	41,260	**11.85 (11.70–12.01)**	**2.74 (2.72)**	**1.27 (0.88)**	**2.31 (2.16)**	**2.72 (2.68)**	**3.08 (3.05)**	**3.47 (3.44)**
Ad5-vectored COVID-19 vaccines	1,266,446	5,681	**3.05 (2.97–3.14)**	**1.54 (1.50)**	1.01 (−0.76)	−0.19 (−2.78)	**0.96 (0.86)**	**2.02 (1.96)**	**2.56 (2.48)**
Inactivated whole-virus COVID-19 vaccines	162,549	93	0.37 (0.30–0.45)	−1.42 (−1.77)	0.25 (−3.53)	N/A	−1.47 (−2.09)	−1.56 (−2.27)	0.00 (−0.57)
Others^*^	60,025	123	**1.60 (1.34–1.91)**	**0.67 (0.37)**	N/A	−0.14 (−2.74)	**0.69 (0.19)**	**1.41 (0.88)**	**1.89 (0.99)**

CI, confidence interval; DTaP-IPV-Hib, diphtheria, tetanus toxoids, pertussis, polio, and *Hemophilus influenza* type b; IC, information component; MMR, measles, mumps, and rubella; N/A, not available; ROR, reported odds ratio.

Bold style indicates that the value of IC_0.25_ > 0.00 or the lower end of the ROR 95% CI > 1.00, are statistically significant.

Numbers in bold indicate a statistical significance.

^*^Others included brucellosis, dengue vaccines, Ebola, leptospirosis, plague, rabies, smallpox, tuberculosis, typhus, and yellow fever vaccines.

### Cumulative report analysis

**Fig. 2:**
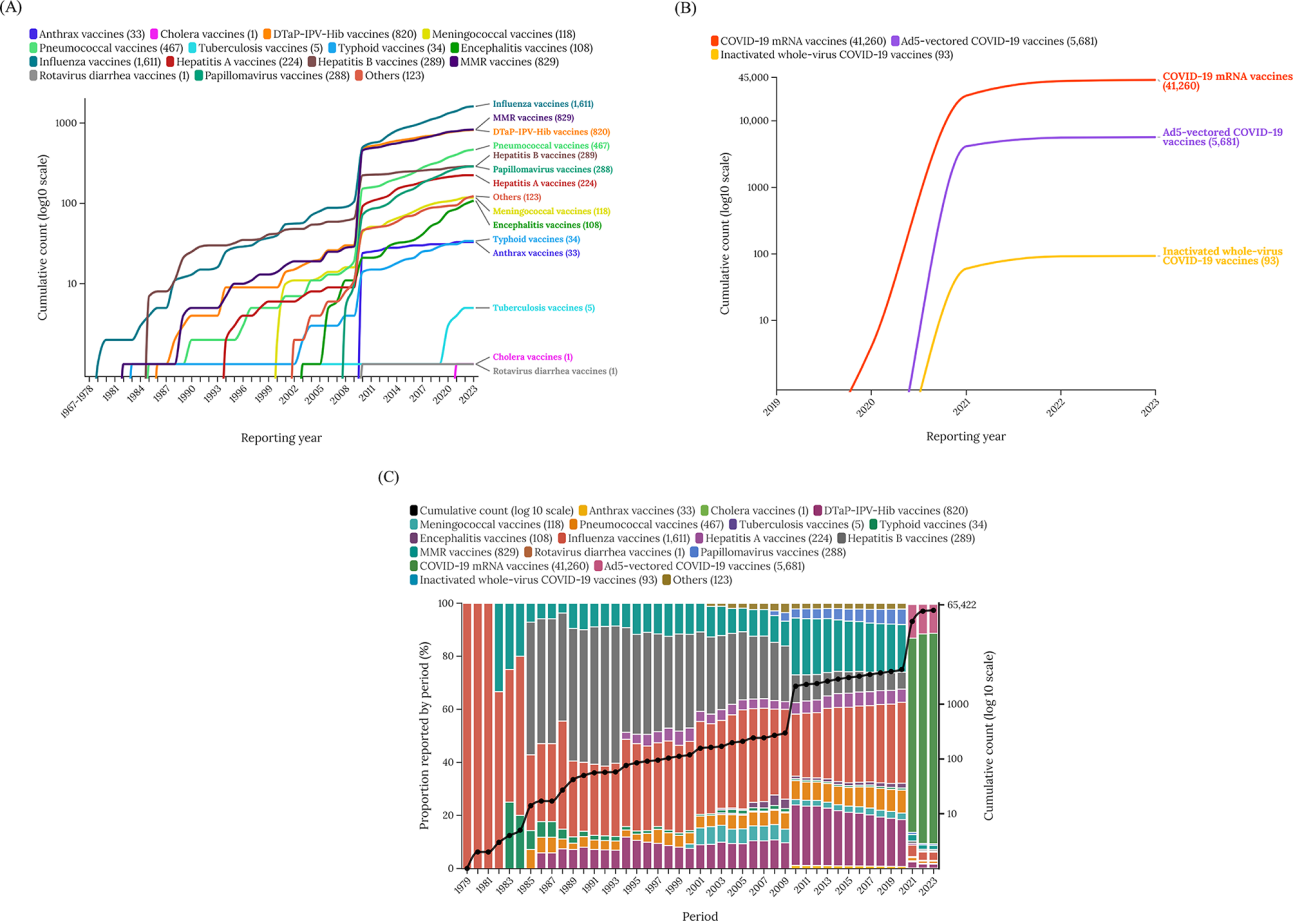
Cumulative number of reports of zoster adverse events per year in association with different vaccines (**A**-**C**). Abbreviation: DTaP-IPV-Hib, diphtheria, tetanus toxoids, pertussis, polio, and *Hemophilus influenza* type b; MMR, measles, mumps, and rubella.

The reporting of vaccine-associated herpes zoster increased significantly from 2011, and a sharp increase can be observed from 2020. From 2011 to 2020, influenza vaccines exhibited a significant association with vaccine-associated herpes zoster. However, since 2020, noticeable influences have also been observed from COVID-19 mRNA vaccines and ad5-vectored COVID-19 vaccines (Fig. 2; Table 3).

**Table 3:** Vaccine class-based description of adverse reactions (heatmap).

	Anthrax vaccines	Cholera vaccines	DTaP-IPV-Hib vaccines	Meningococcal vaccines	Pneumococcal vaccines	Tuberculosis vaccines	Typhoid vaccines	Encephalitis vaccines	Influenza vaccines	Hepatitis A vaccines	Hepatitis B vaccines	MMR vaccines	Rotavirus diarrhea vaccines	Papillomavirus vaccines	COVID-19 mRNA vaccines	Ad5-vectored COVID-19 vaccines	Inactivated whole-virus COVID-19 vaccines	Others^*^
**N observed**	33	1	820	118	467	5	34	108	1,611	224	289	829	1	288	41,260	5,681	93	123
Age, years																		
0–11	0 (0.0)	0 (0.0)	531 (64.8)	34 (28.8)	153 (32.8)	2 (40.0)	1 (2.9)	6 (5.6)	78 (4.8)	119 (53.1)	46 (15.9)	703 (84.8)	1 (100.0)	23 (8.0)	75 (0.2)	4 (0.1)	1 (1.1)	0 (0.0)
12–17	0 (0.0)	0 (0.0)	33 (4.0)	33 (28.0)	3 (0.6)	1 (20.0)	0 (0.0)	6 (5.6)	19 (1.2)	15 (6.7)	21 (7.3)	21 (2.5)	0 (0.0)	151 (52.4)	472 (1.1)	2 (0.0)	0 (0.0)	2 (1.6)
18–44	28 (84.8)	1 (100.0)	67 (8.2)	24 (20.3)	8 (1.7)	2 (40.0)	13 (38.2)	33 (30.6)	208 (12.9)	30 (13.4)	95 (32.9)	31 (3.7)	0 (0.0)	72 (25.0)	9,356 (22.7)	980 (17.3)	29 (31.2)	44 (35.8)
45–64	4 (12.1)	0 (0.0)	98 (12.0)	8 (6.8)	77 (16.5)	0 (0.0)	11 (32.4)	31 (28.7)	526 (32.7)	33 (14.7)	77 (26.6)	21 (2.5)	0 (0.0)	14 (4.9)	13,425 (32.5)	2,526 (44.5)	22 (23.7)	39 (31.7)
≥ 65	1 (3.0)	0 (0.0)	49 (6.0)	6 (5.1)	182 (39.0)	0 (0.0)	6 (17.6)	16 (14.8)	673 (41.8)	15 (6.7)	18 (6.2)	7 (0.8)	0 (0.0)	1 (0.3)	11,904 (28.9)	1,713 (30.2)	35 (37.6)	14 (11.4)
Sex																		
Male	28 (84.8)	1 (100.0)	393 (47.9)	45 (38.1)	222 (47.5)	2 (40.0)	14 (41.2)	55 (50.9)	601 (37.3)	114 (50.9)	119 (41.2)	409 (49.3)	0 (0.0)	28 (9.7)	14,199 (34.4)	1,931 (34.0)	48 (51.6)	66 (53.7)
Female	5 (15.2)	0 (0.0)	422 (51.5)	72 (61.0)	244 (52.2)	3 (60.0)	19 (55.9)	44 (40.7)	992 (61.6)	109 (48.7)	162 (56.1)	410 (49.5)	1 (100.0)	254 (88.2)	26,694 (64.7)	3,612 (63.6)	44 (47.3)	55 (44.7)
**Time to onset**,** days**	1 (1–1)	1 (1–1)	1 (1–1)	1 (1–1)	1 (1–1)	1 (1–1)	1 (1–1)	1 (1–1)	1 (1–1)	1 (1–1)	1 (1–1)	1(1–1)	1(1–1)	1(1–1)	1(1–1)	1(1–1)	1 (1–1)	1(1–1)
**Fatals**	0 (0.0)	0 (0.0)	2 (0.2)	1 (0.8)	1 (0.2)	0 (0.0)	0 (0.0)	0 (0.0)	2 (0.1)	1 (0.4)	2 (0.7)	0 (0.0)	0 (0.0)	0 (0.0)	23 (0.1)	1 (0.0)	0 (0.0)	0 (0.0)
Concomitant adverse events, %																		
Coronary	6.1	0.0	0.4	0.8	1.3	0.0	2.9	0.0	1.4	0.0	2.8	0.2	0.0	1.4	1.1	1.0	1.1	0.8
Arrhythmia	9.1	0.0	2.2	1.7	3.6	20.0	2.9	4.6	3.4	3.1	4.8	2.1	0.0	8.0	3.6	2.6	1.1	6.5
Heart failure	6.1	0.0	0.7	0.8	1.1	0.0	0.0	0.9	0.6	0.4	1.7	0.6	0.0	1.4	0.9	0.3	1.1	0.8
Other cardiac diseases	0.0	0.0	0.0	0.0	0.9	0.0	0.0	0.0	0.4	0.4	0.3	0.1	0.0	0.7	0.6	0.5	1.1	0.8
Hyperthermia	3.0	0.0	6.8	1.7	7.9	20.0	2.9	1.9	3.2	6.7	7.6	7.7	0.0	3.8	3.2	2.7	1.1	8.1
Eosinophilia	0.0	0.0	0.0	0.0	0.0	0.0	0.0	0.0	0.0	0.0	0.0	0.0	0.0	0.0	0.1	0.0	0.0	0.0
Thrombocytopenia and leucopenia	0.0	0.0	0.6	0.0	0.6	20.0	0.0	0.0	0.2	0.4	0.7	0.5	0.0	0.3	0.1	0.2	0.0	0.8
Pulmonary	0.0	0.0	2.0	1.7	1.7	0.0	0.0	0.0	1.7	3.1	2.1	1.6	0.0	1.7	0.7	0.6	0.0	0.0
Infections	93.9	100.0	70.6	81.4	85.9	80.0	88.2	67.6	81.5	94.6	93.4	90.5	100.0	62.8	58.6	34.2	69.9	65.9
Abdominala	3.0	0.0	2.0	3.4	0.6	0.0	0.0	0.0	2.6	3.1	5.2	1.3	0.0	5.2	1.7	1.0	2.2	0.8
Hepato-biliary	0.0	0.0	0.6	0.8	1.1	0.0	0.0	0.0	0.2	2.7	1.7	0.5	0.0	1.0	0.2	0.1	2.2	1.6
Renal	3.0	0.0	0.4	0.0	1.1	0.0	0.0	0.0	1.0	0.9	0.7	0.4	0.0	1.7	0.3	0.1	0.0	0.0
Endocrine	6.1	0.0	0.2	0.8	1.3	0.0	0.0	0.0	0.7	1.3	1.7	0.2	0.0	1.4	0.5	0.4	0.0	1.6
Muscular	18.2	0.0	2.7	3.4	4.5	0.0	2.9	2.8	4.1	4.9	5.2	1.1	0.0	6.6	3.2	2.8	3.2	4.9
Neurologic	36.4	0.0	10.5	12.7	13.9	20.0	5.9	5.6	14.8	14.7	18.0	7.7	0.0	19.8	14.1	14.3	7.5	17.1
Psychiatric	15.2	0.0	1.7	0.8	3.2	0.0	0.0	0.0	3.0	3.6	1.7	1.8	0.0	8.3	1.6	1.0	0.0	2.4
Osteoarticular and rheumatologic	15.2	0.0	1.2	0.0	3.9	0.0	0.0	0.9	2.5	0.9	5.2	1.0	0.0	4.9	2.2	1.5	2.2	2.4
Dermatologic	27.3	100.0	29.0	22.0	33.4	60.0	35.3	9.3	27.6	31.7	32.2	30.5	0.0	19.4	16.7	11.9	3.2	22.8
Anaphylaxis	0.0	0.0	0.4	1.7	0.2	0.0	0.0	0.9	0.7	0.4	1.0	0.1	0.0	0.0	0.3	0.1	0.0	0.0
Ophthalmology	9.1	0.0	1.6	0.0	3.4	0.0	0.0	0.9	1.9	2.2	1.7	0.8	0.0	2.1	1.1	0.5	0.0	4.1

The heatmap was expressed according to the percentage value.

DTaP-IPV-Hib, diphtheria, tetanus toxoids, pertussis, polio, and *Hemophilus influenza* type b; IQR, interquartile range; MMR, measles, mumps, and rubella; TTO, time to onset.

^*^Others included brucellosis, dengue vaccines, Ebola, leptospirosis, plague, rabies, smallpox, tuberculosis, typhus, and yellow fever vaccines.

## Discussion

### Main findings

This is the first study to investigate the potential association between various vaccines and herpes zoster across a large population (total = 7,805,380) over a long period, from 1969 to 2023. The reporting of vaccine-associated herpes zoster has sharply increased since the introduction of influenza vaccines in 2011, with a further rapid rise observed in 2020 following the introduction of COVID-19 mRNA vaccines and ad5-vectored COVID-19 vaccines. The COVID-19 mRNA vaccine showed the greatest ROR and IC value with vaccine-associated herpes zoster, followed by the encephalitis vaccine, influenza vaccine, and ad5-vectored COVID-19 vaccine. Regarding sex and age, an increased rate of ROR and IC for vaccine-associated herpes zoster was observed among males and older populations. While zoster can lead to various complications and further research is needed for the appropriate management of vaccine-associated herpes zoster, its mortality rate is low. Therefore, there is no need to hesitate about vaccination.

### Interpretations of the study findings

Our study found that COVID-19 mRNA vaccines and ad5-vectored COVID-19 vaccines are significantly associated with vaccine-associated herpes zoster. However, according to previous research, the degree of association varied between these two, which can be attributed to differences in cytokine activation and T-cell immune responses among vaccines. The mechanism by which these vaccines might trigger herpes zoster involves the role of T lymphocytes in controlling VZV during its latent phase. Vaccination-induced T-cell dysfunction could play a part in this occurrence. Presenting more detailed mechanisms, it has been observed that COVID-19 vaccination induces a robust T-cell response and enhances the proinflammatory action profile in potent antigen-presenting cells via the spike protein, potentially causing VZV reactivation. Additionally, a significant shift of naive CD8 + cells to create specific CD8 + cells might temporarily impair the control of VZV by VZV-specific CD8 + cells. Furthermore, changes in Toll-like receptor signaling due to vaccination or the effects of cytokines such as type I interferon might be involved^9^.

Another vaccine that showed an association with vaccine-associated herpes zoster in the present study is the encephalitis vaccine. There have been reports of systemic urticaria or rash following encephalitis vaccination^21^. Additionally, side effects such as fatigue suggest the possibility of zoster occurrence due to immunosuppression. Influenza vaccines are hypothesized to cause vaccine-associated herpes zoster due to the dysregulation of T cells and cellular immunity. This dysregulation is likely due to immune modulation induced by the vaccines’ target vectors or adjuvants, which could play a role in reactivating the virus. It is also speculated that the reactivation of VZV is caused by the interaction of various factors with a summation effect^22^.

Our research indicates that the value of ROR and IC for vaccine-associated zoster due to vaccines is higher in males. Although, previous studies have reported either a higher incidence of zoster in females or no significant difference based on sex, there has been no precise analysis explaining these results. Additionally, it has been suggested that this discrepancy could be due to females being more likely to visit their clinics when experiencing symptoms of zoster^23–25^. Therefore, further research is needed to understand why the risk varies between sexes.

### Comparison of previous studies

Previous studies investigated the impact of COVID-19 mRNA, ad5-vectored COVID-19, and influenza vaccines on vaccine-associated herpes zoster, showing results similar to ours^6,9,22,26^. However, these studies were limited by their short duration and smaller sample sizes, which lowered their reliability. In contrast, our study was conducted over a more extended period with a larger population, encompassing all types of vaccines. Additionally, by targeting a global population, we further increased the credibility of our results.

Other prior research has indicated that females and older individuals are more susceptible to zoster. While prior studies have investigated the association between age, sex, and the onset of herpes zoster^27^, there has been no investigation into how vaccine influences change depending on age and sex. Therefore, this study is the first to explore how vaccines influence zoster across different age groups and sexes.

### Clinical policy implications

While herpes zoster is not a disease with a high mortality rate^28^, it is a quality-of-life-threatening condition characterized by very painful rashes and neuralgia^29^. It can also carry the risk of leading to severe complications^30^. Moreover, there are ongoing reports regarding vaccine-associated herpes zoster. Despite this, there is a lack of research on vaccine-associated herpes zoster. Therefore, it is imperative to facilitate more related studies and use the accumulated results from various research to propose compensation and management manuals.

Vaccine-associated herpes zoster occurs when the latent VZV virus reactivates, primarily controlled by T lymphocytes. As the dysregulation of T cells is the leading cause of vaccine-associated herpes zoster, an individual’s immune status plays a crucial role^31^. Consequently, it is essential to properly assess an individual’s health status before administering a vaccine and provide guidance accordingly. This study also found that older individuals are especially susceptible to vaccine-associated herpes zoster. Therefore, checking a patient’s immune status before vaccination is necessary, and attention should be paid to older individuals and males.

### Strengths and limitations

Using a global pharmacovigilance database, this study is the first to conduct a long-term investigation on the side effects of zoster associated with various vaccines across a broad population. Furthermore, this research also observed how these side effects vary according to age and sex, thereby enhancing the reliability of the findings compared to previous studies. However, despite these strengths, the present study still has several limitations.

First, due to the inherent characteristics of the study being a cross-sectional analysis, a limitation arises because it is not possible to definitively ascertain the causes of the outcomes derived from the data analysis within the results themselves^32^. However, the study attempted to explain the mechanisms as clearly as possible, building upon previous findings. Furthermore, if future studies are conducted to analyze the causes based on the results, the understanding of vaccine-associated herpes zoster could be significantly advanced.

Second, the structure of the reporting system that analyzes vaccine-associated herpes zoster has limitations. This study relied on passive reporting methods, which may lead to an underrepresentation of vaccine-associated herpes zoster cases and distort report rates. Therefore, it is probable that fewer incidents were reported than occurred, suggesting the possibility that associations were observed less than they exist.

Third, there may be bias in the results due to the possibility that individuals who received vaccines managed their health and well-being better than those who did not receive vaccinations. This is a consequence of not being able to exclude the various characteristics possessed by the vaccinated individuals, potentially leading to errors attributable to the specific environments of certain groups. However, we included a large number of participants, and all values utilized in interpreting the research findings were significant. Therefore, these factors are unlikely to have significantly reduced the reliability of the results.

Fourth, the lack of reports on live attenuated vaccines among encephalitis vaccines in this study limited a comprehensive analysis of their effects. Although the study was initially designed to include an analysis of live attenuated vaccines, there was insufficient data on live attenuated vaccines related to vaccine-associated herpes zoster, preventing their inclusion in the study. Consequently, the findings related to encephalitis vaccines in this study should primarily be interpreted based on inactivated vaccines, and further comprehensive research that includes live attenuated vaccines is warranted. Fifth, various reporting biases may have influenced the study results. In particular, this study observed a sharp increase in reporting rates after 2011, which may have been influenced by the development of reporting systems. Additionally, in countries with well-established reporting systems, vaccine-related adverse events are more likely to be reported compared to those with less developed systems. Therefore, interpreting an increase in reported cases solely as a rise in adverse event rates may not be appropriate, and cautious interpretation and thorough examination are required when considering the generalizability of the findings. Lastly, our analysis was based on self-reported data obtained from the WHO global pharmacovigilance database. Due to the inability to determine the population at risk required for incidence rate calculation, we were unable to analyze the incidence rate of vaccine-associated herpes zoster.

## Conclusion

This study is the first to observe the global impact of various vaccines on herpes zoster over a long period, from 1969 to 2023. Vaccine-associated herpes zoster increased due to the influenza vaccines starting in 2011, with a sharp rise after the introduction of COVID-19 vaccines in 2020. Although various vaccines can lead to herpes zoster, the mortality rate is very low, so vaccination might not be hesitated. The highest ROR and IC values for vaccine-associated herpes zoster were observed with the COVID-19 mRNA vaccine, followed by the encephalitis vaccine, influenza vaccine, and ad5-vectored COVID-19 vaccine. Additionally, this study identified elevated ROR and IC rates for vaccine-associated herpes zoster among males and older individuals. Based on the research findings, it is essential to consider the immune status of individuals receiving vaccines and to implement appropriate compensation and management manuals for vaccine-associated herpes zoster.

## Electronic supplementary material

Below is the link to the electronic supplementary material.

Supplementary Material 1

## Data Availability

The data are available upon request. Study protocol and statistical code: Available from DKY (yonkkang@gmail.com). Dataset: The data used in this study are not publicly available. Available from the Uppsala Monitoring Centre or World Health Organization through a data use agreement.
