# Supplementary material for: Global and regional estimates of vaccine-associated herpes zoster and their related vaccines from 1969 to 2023
Source: Sci Rep. 2025 Apr 17;15:13285. doi: 10.1038/s41598-025-98106-9 (PMC12006434; doi:10.1038/s41598-025-98106-9)
Supplement: Supplementary file 1 — Supplementary Material 1 [file 41598_2025_98106_MOESM1_ESM.docx]

| Supplementary materials |
| --- |

Original Article

Global and regional estimates of vaccine-associated herpes zoster and their related vaccines from 1969 to 2023

Running title: Vaccine-associated herpes zoster

Jinyoung Jeong,^1,2&^ Hyesu Jo,^2,3&^ Yejun Son,^2,4&^ Jaeyu Park,^2,4&^ Jiyeon Oh,^1,2^ Sooji Lee,^1,2^ Yi Deun Jeong,^1,2^ Kyeongmin Lee,^2,3^ Hyeon Jin Kim,^2,4^ Hayeon Lee,^2,5^ Soeun Kim,^2,4^ Yesol Yim,^2,4^ Masoud Rahmati,^6,7,8^ Jiseung Kang,^9,10^ Raphael Udeh,^11^ Damiano Pizzol,^12,13^ Lee Smith,^14*^ Jiyoung Hwang,^2*^ Dong Keon Yon,^1,2,3,4,15*^

^&^ These authors contributed equally as co-first author

^*^ These authors contributed equally as the corresponding author

**^*^Corresponding authors**

**Lee Smith,** PhD

Centre for Health, Performance and Wellbeing, Anglia Ruskin University, Cambridge CB1 1PT, UK

Email: Lee.Smith@aru.ac.uk

**Jiyoung Hwang**, PhD

Center for Digital Health, Medical Science Research Institute Kyung Hee University Medical Center, Kyung Hee University College of Medicine, 23 Kyungheedae-ro, Dongdaemun-gu, Seoul, 02447, South Korea

E-mail: cindy.jyhwang@gmail.com

**Dong Keon Yon**, MD, PhD, FACAAI, FAAAAI, ATSF

Department of Pediatrics, Kyung Hee University College of Medicine, 23 Kyungheedae-ro, Dongdaemun-gu, Seoul, 02447, South Korea

Email: yonkkang@gmail.com

**Table S1.** Medical Dictionary for Regulatory Activities (MedDRA) preferred terms and classifications for zoster.

| SOC | HLGT | HLT | PT | LLT | MedDRA code |
| --- | --- | --- | --- | --- | --- |
| Infections and infestations | Viral infectious disorders | Herpes viral infections | Herpes zoster | Acute posterior ganglionitis | 10081165 |
| Infections and infestations | Viral infectious disorders | Herpes viral infections | Herpes zoster | Herpes zoster | 10019974 |
| Infections and infestations | Viral infectious disorders | Herpes viral infections | Herpes zoster | Herpes zoster (excl ophthalmic) | 10019975 |
| Infections and infestations | Viral infectious disorders | Herpes viral infections | Herpes zoster | Herpes zoster C2 | 10019976 |
| Infections and infestations | Viral infectious disorders | Herpes viral infections | Herpes zoster | Herpes zoster dermatitis | 10019977 |
| Infections and infestations | Viral infectious disorders | Herpes viral infections | Herpes zoster | Herpes zoster NOS | 10019982 |
| Infections and infestations | Viral infectious disorders | Herpes viral infections | Herpes zoster | Herpes zoster with other specified complication | 10019990 |
| Infections and infestations | Viral infectious disorders | Herpes viral infections | Herpes zoster | Herpes zoster with other specified complications | 10019991 |
| Infections and infestations | Viral infectious disorders | Herpes viral infections | Herpes zoster | Herpes zoster with unspecified complication | 10019993 |
| Infections and infestations | Viral infectious disorders | Herpes viral infections | Herpes zoster | Herpes zoster without mention of complication | 10019995 |
| Infections and infestations | Viral infectious disorders | Herpes viral infections | Herpes zoster | Shingles | 10040555 |
| Infections and infestations | Viral infectious disorders | Herpes viral infections | Herpes zoster | Zona | 10067526 |
| Infections and infestations | Viral infectious disorders | Herpes viral infections | Herpes zoster cutaneous disseminated | Herpes zoster bilateral | 10074299 |
| Infections and infestations | Viral infectious disorders | Herpes viral infections | Herpes zoster cutaneous disseminated | Herpes zoster cutaneous disseminated | 10074297 |
| Infections and infestations | Viral infectious disorders | Herpes viral infections | Herpes zoster cutaneous disseminated | Herpes zoster multi-dermatomal | 10058428 |
| Infections and infestations | Viral infectious disorders | Herpes viral infections | Herpes zoster reactivation | Herpes zoster reactivation | 10080516 |
| Infections and infestations | Viral infectious disorders | Herpes viral infections | Genital herpes zoster | Genital herpes zoster | 10072210 |
| Infections and infestations | Viral infectious disorders | Herpes viral infections | Genital herpes zoster | Genital zoster | 10072218 |
| Infections and infestations | Viral infectious disorders | Herpes viral infections | Genital herpes zoster | Perianal herpes zoster | 10080101 |
| Infections and infestations | Viral infectious disorders | Herpes viral infections | Ophthalmic herpes zoster | Herpes zoster dermatitis of eyelid | 10019978 |
| Infections and infestations | Viral infectious disorders | Herpes viral infections | Ophthalmic herpes zoster | Herpes zoster iridocyclitis | 10019980 |
| Infections and infestations | Viral infectious disorders | Herpes viral infections | Ophthalmic herpes zoster | Herpes zoster keratitis | 10073932 |
| Infections and infestations | Viral infectious disorders | Herpes viral infections | Ophthalmic herpes zoster | Herpes zoster keratoconjunctivitis | 10019981 |
| Infections and infestations | Viral infectious disorders | Herpes viral infections | Ophthalmic herpes zoster | Herpes zoster ophthalmic | 10019983 |
| Infections and infestations | Viral infectious disorders | Herpes viral infections | Ophthalmic herpes zoster | Herpes zoster ophthalmicus | 10019984 |
| Infections and infestations | Viral infectious disorders | Herpes viral infections | Ophthalmic herpes zoster | Herpes zoster with ophthalmic complications | 10019987 |
| Infections and infestations | Viral infectious disorders | Herpes viral infections | Ophthalmic herpes zoster | Herpes zoster with other ophthalmic complications | 10019989 |
| Infections and infestations | Viral infectious disorders | Herpes viral infections | Ophthalmic herpes zoster | Ophthalmic herpes zoster | 10030865 |
| Infections and infestations | Viral infectious disorders | Herpes viral infections | Ophthalmic herpes zoster | Ophthalmic zoster | 10030868 |
| Infections and infestations | Viral infectious disorders | Herpes viral infections | Herpes zoster oticus | Geniculate herpes | 10018140 |
| Infections and infestations | Viral infectious disorders | Herpes viral infections | Herpes zoster oticus | Geniculate herpes zoster | 10018141 |
| Infections and infestations | Viral infectious disorders | Herpes viral infections | Herpes zoster oticus | Herpes zoster oticus | 10063491 |
| Infections and infestations | Viral infectious disorders | Herpes viral infections | Herpes zoster oticus | Herpes zoster otitis externa | 10019985 |
| Infections and infestations | Viral infectious disorders | Herpes viral infections | Herpes zoster oticus | Otitis externa due to herpes zoster | 10033077 |
| Infections and infestations | Viral infectious disorders | Herpes viral infections | Herpes zoster oticus | Ramsay-Hunt syndrome | 10037834 |
| Infections and infestations | Viral infectious disorders | Herpes viral infections | Herpes zoster infection neurological | Herpes zoster infection neurological | 10061208 |
| Infections and infestations | Viral infectious disorders | Herpes viral infections | Herpes zoster infection neurological | Herpes zoster infection neurological NOS | 10019979 |
| Infections and infestations | Viral infectious disorders | Herpes viral infections | Herpes zoster infection neurological | Herpes zoster with other nervous system complications | 10019988 |
| Infections and infestations | Viral infectious disorders | Herpes viral infections | Herpes zoster infection neurological | Herpes zoster with other specified nervous system complication | 10019992 |
| Infections and infestations | Viral infectious disorders | Herpes viral infections | Herpes zoster infection neurological | Herpes zoster with unspecified nervous system complication | 10019994 |
| Infections and infestations | Viral infectious disorders | Herpes viral infections | Herpes zoster meningitis | Herpes zoster meningitis | 10074259 |
| Infections and infestations | Viral infectious disorders | Herpes viral infections | Herpes zoster meningitis | Herpes zoster with meningitis | 10019986 |
| Infections and infestations | Viral infectious disorders | Herpes viral infections | Herpes zoster meningitis | Meningitis due to herpes zoster virus | 10027218 |
| Infections and infestations | Viral infectious disorders | Herpes viral infections | Herpes zoster meningoencephalitis | Herpes zoster meningoencephalitis | 10074248 |
| Infections and infestations | Viral infectious disorders | Herpes viral infections | Herpes zoster meningoencephalitis | Varicella zoster encephalitis | 10074255 |
| Infections and infestations | Viral infectious disorders | Herpes viral infections | Herpes zoster meningomyelitis | Herpes zoster meningomyelitis | 10074251 |
| Infections and infestations | Viral infectious disorders | Herpes viral infections | Herpes zoster meningomyelitis | Herpes zoster myelitis | 10074258 |

 DRESS, drug reaction with eosinophilia and systemic symptoms; HLT, high-level term; HLGT, high-level group term; LLT, lower-level terms; PT, preferred terms; SOC, system organ class
